# Neolithic hydroclimatic change and water resources exploitation in the Fertile Crescent

**DOI:** 10.1038/s41598-022-27166-y

**Published:** 2023-01-13

**Authors:** Eleonora Regattieri, Luca Forti, Russell N. Drysdale, Giorgio Mannella, John C. Hellstrom, Cecilia Conati Barbaro, Daniele Morandi Bonacossi, Andrea Zerboni

**Affiliations:** 1 Istituto di Geoscienze e Georisorse, IGG-CNR, Via Moruzzi 1, 56126 Pisa, Italy; 2 Istituto Nazionale di Geofisica e Vulcanologia INGV, Pisa, Italy; 3 Dipartimento di Scienze delle Terra “A. Desio”, Università degli Studi di Milano, Via L. Mangiagalli 34, 20133 Milan, Italy; 4 School of Geography, Earth and Atmospheric Sciences, University of Melbourne, Melbourne, Parkville, 3010 VIC Australia; 5 Dipartimento di Scienze della Terra, Università di Pisa, 56126 Pisa, Italy; 6 Dipartimento di Scienze dell’Antichità, Università di Roma Sapienza, 00185 Rome, Italy; 7 Dipartimento di Studi Umanistici e del Patrimonio Culturale, Università di Udine, 33100 Udine, Italy

**Keywords:** Palaeoclimate, Archaeology

## Abstract

In the first millennia of the Holocene, human communities in the Fertile Crescent experienced drastic cultural and technological transformations that modified social and human-environments interactions, ultimately leading to the rise of complex societies. The potential influence of climate on this “Neolithic Revolution” has long been debated. Here we present a speleothem record from the Kurdistan Region of Iraq, covering from Early Neolithic to Early Chalcolithic periods (~ 11 to 7.3 ka, 9000–5300 BCE). The record reveals the influence of the Siberian High on regional precipitation, and shows large hydroclimatic variability at the multicentennial scale. In particular, it highlights wetter conditions between 9.7 and 9.0 ka, followed by an abrupt reduction of precipitation between 9.0 and 8.5 ka, and a wetter interval between 8.5 and 8.0 ka. A comparison with regional and local archaeological data demonstrates an influence of recorded hydroclimatic changes on settlement patterns (size, distribution, permanent vs. seasonal occupation) and on the exploitation of water resources by Neolithic to Chalcolithic populations. Our record does not show prominent hydroclimatic changes at 9.3 and 8.2 ka, thus not supporting direct influence of such rapid and widespread events on the process of Neolithization and its cultural dispersal.

## Introduction

During the first half of the Holocene (11.6–7 ka, 9600–5000 BCE), human communities across SW Asia developed new strategies for living as sedentary farmers and herders following millennia of a mobile hunter-gathering existence^1,2^. This so-called “Neolithic Revolution” was a progressive and spatially nuanced process that involved fundamental social, economic, and technological transformations, resulting in the aggregation of people into permanent settlements^3^, the progressive domestication of animal and plant species^4,5^, and dramatic population growth^6^. Such innovations reorganized human–environment and social interactions, ultimately leading to the rise of complex societies^7,8^. Detailed records of climate variability and its underlying mechanisms from these nascent regions are crucial to fully understand the environmental context of the Neolithic transition. In particular, such records can shed light on the potential nexus between climatic and environmental change on the one hand, and local cultural trajectories, demographic trends, and the dispersal of farming practices on the other.

The first part of the Holocene was also punctuated by multidecadal- to centennial-scale rapid climate changes (or RCCs) of hemispheric-to-global scale which are characterized by a high degree of temporal and spatial variability, and by complex and non-stationary patterns of regional climate teleconnections^9^. Two major centennial-scale intervals of abrupt climate change have been recognized globally around 9.3 ka and 8.2 ka^10,11^ with the latter being the most prominent and widespread^12,13^. These events were triggered by drainage of freshwater from the last remnants of the Laurentide ice sheet to the North Atlantic, and caused short-term weakening of the North Atlantic overturning circulation (AMOC), with downstream effects on oceanic and atmospheric conditions^14,15^. Given their pervasive influence and their significance at the scale of humans and ecosystems, their expression in SW Asia has long been investigated to identify potential links with Neolithic phases of socio-economic transformations^16,17^. Yet, contrasting evidence exists regarding the local impacts of these events, with some studies suggesting they were associated with droughts—especially at 8.2 ka^16^ whilst others pointing to a muted or even wetter climate, sometimes within longer intervals of particularly unstable conditions^18,19^. Even more debated is the influence of such climatic events on Neolithic populations and their adaptive response, with some scholars identifying major cultural outbreaks at the time, and others suggest more complex scenarios of resilience and cultural adaptation^16,17^.

Geochemical records from cave chemical deposits (speleothems) are ideally suited for comparison with contextually robust evidence from modern archaeological and historical studies, because they preserve multiple environmentally sensitive properties that can be attributed to regional and extra-regional climatic forcing^20–22^. Speleothems can also be precisely dated by the U–Th disequilibrium method, providing an independent time frame by which to interrogate the triggers and propagation mechanisms of change^23^. Here, we present a multi-decadal hydroclimatic reconstruction spanning the 11.0–7.6 ka period (9000–5600 years BCE) obtained from a speleothem from the Zagros Mountains piedmont zone of Iraqi Kurdistan, located in the core region of the Fertile Crescent (FC). Current research along the Zagros high steppe and foothills indicates the region was a key zone in the Neolithic transition^3^. Ancient DNA evidence suggests that the area experienced an independent trajectory from hunter gathering to cultivation of cereal crops and animal husbandry^4,5,24^, while archaeological research indicates that it hosted some of the earliest, large and permanent settlements^3^, and some of the earliest evidence for pottery production^25^. The speleothem record presented here encompasses the local cultural entities of the Early Neolithic (Pre-Pottery Neolithic or PPN 11.6–9.0 ka/9600–7000 years BCE), Late Neolithic (Pottery Neolithic or PN 9–7.3 ka/7000–5300 years BCE), and the Early Chalcolithic (7.3–6.5 ka, 5300–4500 years BCE)^3,26^. Throughout these periods, local communities underwent profound transformations in material cultures and subsistence strategies, as well as marked changes in settlement patterns^3,26^. The speleothem records multi-centennial changes in regional moisture availability, reaching their maximum amplitude between 9.5 and 8.0 ka (7500–6000 BCE). A comparison with regional and extra-regional records reveals that the North Atlantic-triggered 9.3 and 8.2 ka events had only a limited impact on the local climate, and that hydrologic variability in the northern FC was mostly affected by changes in the intensity of the Siberian High (SH). The SH, the dominant semi-permanent anticyclone that occurs during winter-early spring and controls temperature and precipitation in large parts of Asia^27,28^, triggered changes in the amount and style of precipitation, and modulated the length of the wet season in the northern FC. By exploring the temporal relationship with the major cultural transitions apparent from the local archaeological record, we found that hydroclimatic fluctuations are coeval with major changes in settlement’s pattern and re-organization of the strategies for the exploitation of water resources. This evidence suggests a link between hydroclimatic variability, social organization and human exploitation of the environment in the Neolithic and at the Neolithic-Chalcolithic transition.

## Site settings

**Figure 1:**
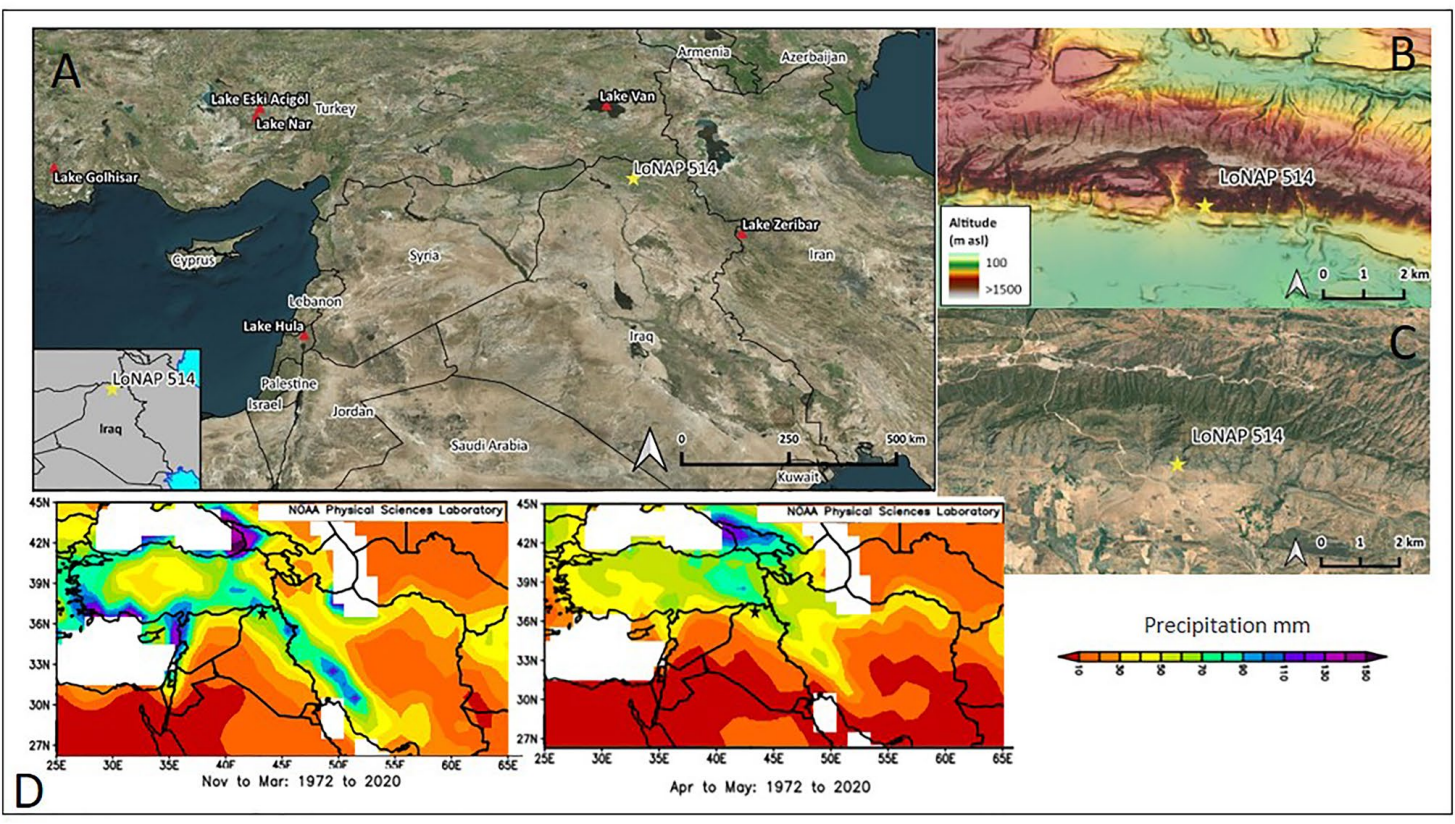
(**A**) Map of the Fertile Crescent region showing the location of the study site (LoNAP 514) and the main regional paleoclimate archives discussed in the text; (**B**, **C**) (elaborated with QGIS 3.16.7 plugin https://nextgis.com/blog/quickmapservices/); (**B**, **C**) Digital elevation model (DEM) and satellite imagery of the northern Kurdistan region of Iraq (DEM free downloaded from https://www.eorc.jaxa.jp/ALOS/en/aw3d30/ and elaborated with QGIS 3.16.7; satellite imagery elaborated with QGIS 3.16.7 plugin https://nextgis.com/blog/quickmapservices/); (**D**) Maps of mean monthly precipitation in winter and spring (downloaded from https://psl.noaa.gov/). The star is the LoNAP514 site.

The speleothem studied here was collected during the archaeological survey carried out in the northern Kurdistan region of Iraq by the “Land of Ninive Archaeological Project” (LoNAP, see Acknowledgement), from a shallow, unnamed cave registered as 514 in the record of the survey (Fig. 1). Therefore, the specimen is named LoNAP514 hereinafter. The cave (~ 940 m a.s.l.) formed in the Paleogene limestones of the SW foothills of the Zagros Mountains, facing the alluvial plains formed by the left bank tributaries of the Tigris River^29^ (Fig. 1, and Supplementary Information text-1). Total annual precipitation at the cave site, based on 0.5° grid cell re-analysis data, averages ~ 600 mm (1901–2016)^30^ and is higher than in the southern semi-arid part of the country due to local orographic effects. About 98% of the precipitation occurs between November and May^31^. There is an overall influence of westerly circulation, with most of the precipitation events associated with storm tracks originating in the eastern Mediterranean, especially from the Cyprus low, and following an eastward trajectory. A significant amount of precipitation is also related to strong convective instability associated with low-level water vapor supply^31^. In wet transition months (ON and MAM), southeasterly winds also supply vapor from the Persian Gulf, and the Arabian and Red Seas, increasing convection and contributing significantly to total annual precipitation^32–34^. These low-level, southern humidity fluxes are almost suppressed in dry years^32^. The Black and Caspian Seas contribute little to precipitation in the region^31^. On multidecadal time-scales, low correlation (> 10%) is observed between the inter-annual variability of FC precipitation and the index of the North Atlantic Oscillation^31^, implying that latitudinal shifts and/or changes in the strength of westerly winds do not affect directly precipitation variability in the study area.

## Results and hydroclimatic significance of the record

LoNAP514 was sampled from a flowstone. It is 86 mm thick and composed of compact columnar calcite (Fig. S1). The age-depth model (Fig. S2) is based on 15 U/Th ages (Table S1) and covers the 11.0 ± 0.3 to 6.6 ± 0.2 ka time span; the mean age uncertainty is 140 years. Ages are given in thousands of years (ka) with respect to the year of measurement (2020), and reported in BCE when discussed along with the archaeological information.

**Figure 2:**
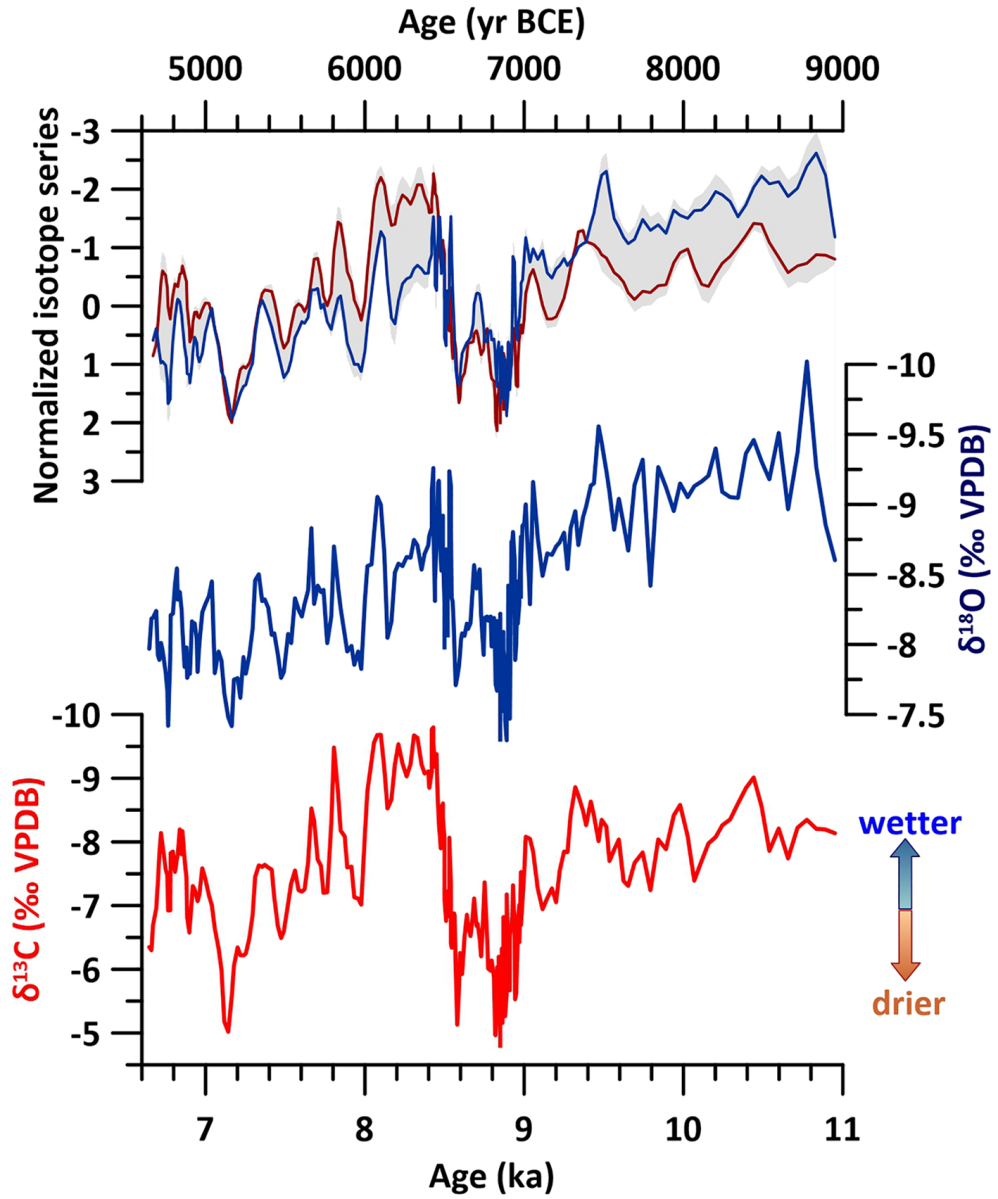
Stable carbon (δ^13^C, red) and oxygen (δ^18^O, blue) isotope series for flowstone LoNAP514; top: 3 points- smoothed and normalized isotope time series (SOM text), the grey band represents the standard deviation between the normalized series (see Supplementary Information).

The speleothem stable carbon and oxygen isotope composition (δ^13^C and δ^18^O, Table S2) time series have a decadal resolution. They are significantly positively correlated (r = 0.69) and show very similar centennial to multi-centennial patterns (Fig. 2 and Supplementary Information text-2).

This indicates a common first-order driver for their coupled variability over these time scales. Assuming quasi-isotopic equilibrium calcite precipitation (Supplementary Information, text 3) and given minimal early-mid Holocene temperature changes inside the cave, the LoNAP514 δ^18^O is a proxy for drip-water δ^18^O, which reflects local meteoric precipitation δ^18^O (δ^18^O_p_). δ^18^O_p_ results from changes in moisture source location and isotopic composition, condensation temperature and seasonality^35^ (Supplementary information text-4 and Fig. S3). Here, we interpret centennial-scale variations in the δ^18^O as primarily recording rainfall fluctuations, with lower/higher δ^18^O ratios occurring during wetter/drier periods respectively (Supplementary information text-4). This interpretation is consistent with speleothem δ^18^O records from central to eastern Mediterranean, the Middle East, and the Arabian Peninsula^36–40^. For LoNAP514, it is also supported by the positive covariance with the δ^13^C time series. In fact, the range of δ^13^C values (− 9.80‰ to − 4.81‰) is indicative of a prevalent input of organic, ^13^C-depleted CO_2_ from the overlying soil, and is typical of a predominantly C3 vegetation, which was likely present in the cave catchment over the period of LoNAP514 deposition (Supplementary Information text-5). In these settings, δ^13^C variations arise from fluctuating soil biological activity, in turn related to temperature and precipitation^41^, with the latter likely predominating for LoNAP514 due to relatively stable Holocene temperatures. Higher rainfall amounts (lower δ^18^O_p_) foster soil metabolism and vegetation development, leading to lower δ^13^C. Under drier conditions (higher δ^18^O_p_), the biogenic CO_2_ supply is reduced, causing higher δ^13^C. The hydrological sensitivity of both δ^18^O and δ^13^C is further increased during drier periods by evaporation at the topsoil, in the epikarst, and degassing at the site of stalagmite deposition. These processes cause preferential loss of lighter C and O isotopes, leading to stronger covariation and increased isotope ratios in speleothem calcite^42^. This is particularly apparent in our record between 9.0 and 8.4 ka and between 7.6 and 7.0 ka, where the isotope series show highest values and the lowest difference between normalized values (Fig. 2). The largest discrepancy between the two series is observed between 10.8 and 9.4 ka, corresponding to the most negative and least variable δ^18^O values. This interval is near-synchronous with depleted δ^18^O values observed in the planktonic foraminifera (*Globigerinoides ruber*) record from marine cores LC21 and SL21 from the central Aegean Sea (Fig. S5), which reflect the inflow of fresh (isotopically depleted) surface waters from the Nile River due to an enhanced African monsoon recharging the upper Nile catchment^43^. Such freshwater influx corresponds to a calculated decrease of ~ 1.3‰ in local surface seawater δ^18^O^43^. Given that the Eastern Mediterranean is one of the main sources of precipitation for our study area^31–34^, this depletion was likely transmitted to our oxygen record via the source effect (Supplementary information, text-4). The latter likely buffered centennial-scale variability due to the rainfall amount effect over this interval, and might result in an internal variability (or absence of it) that is not linked to the amount of precipitation. A prevailing influence of the source effect in our oxygen record in some particular intervals can be also inferred by the comparison between with the Fe/Ca record from an offshore core (MS27PT) located at the Nile mouth^44^, that represents a direct proxy for fluvial terrigenous input related to increased monsoon rain over the upper Nile catchment (Fig. S5). In the MS27PT record, enhanced Nile flow is apparent before 9.4 ka, and centennial-scale terrigenous peaks correspond to negative spikes in the δ^18^O record (e.g. at 8.4 ka, 8.1 ka, Fig. S5), but have no strict correspondence in the carbon record (Fig. S5). Therefore, we consider the δ^13^C as a more robust and direct proxy for moisture changes at the cave site.

**Figure 3:**
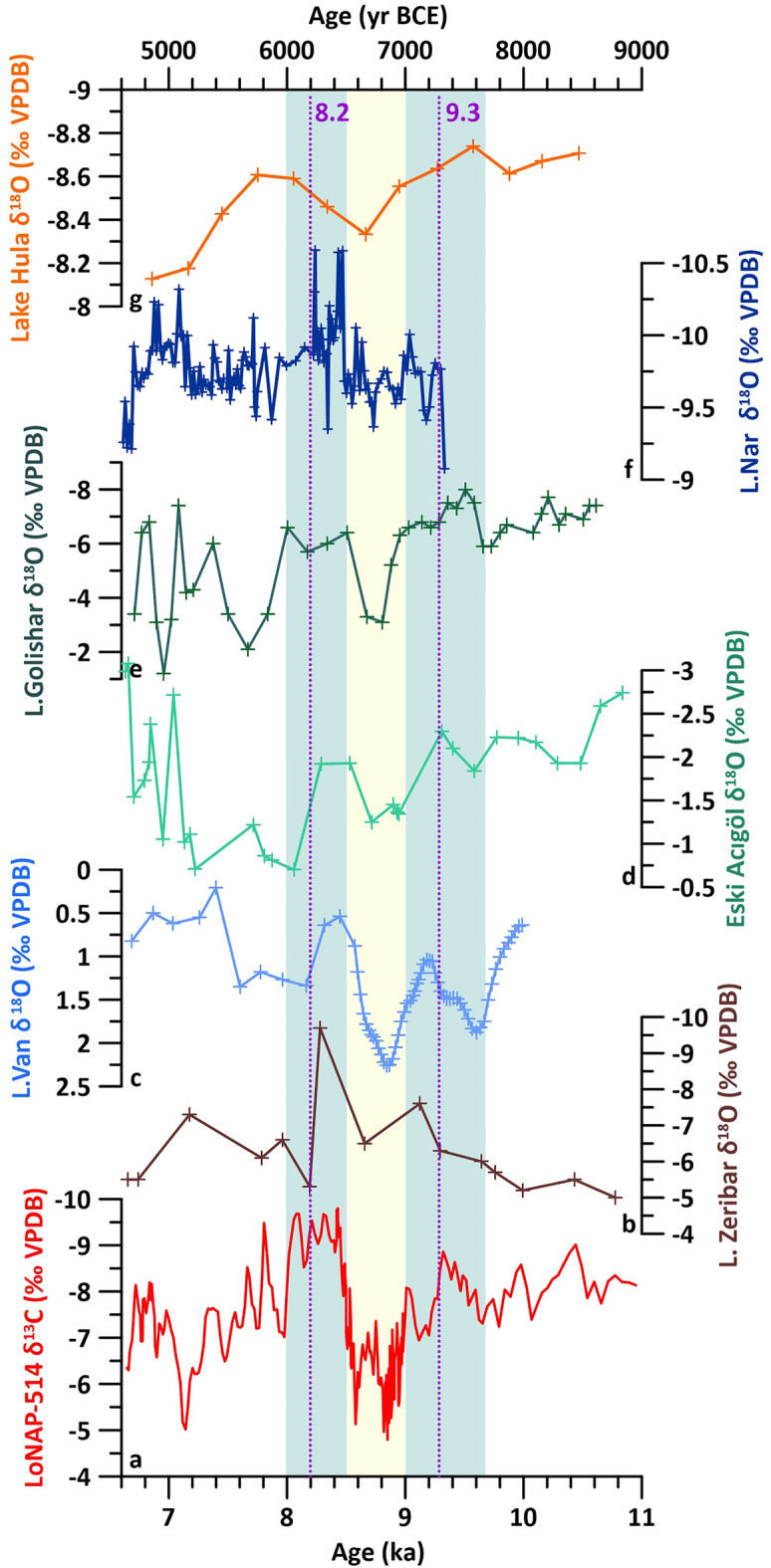
Regional comparison among (**a**) LoNAP514 δ^13^C (this study) and δ^18^O records from (**b**) Lake Zeribar^46^, (**c**) Lake Van^48^, (**d**) Lake Eski Acigöl^47^, (**e**) Lake Golishar^49^, (**f**) Nar Gölü^50^, (**g**) Lake Hula^45^. Blue/yellow shaded areas highlight the common main wetter/drier intervals; dotted lines indicate the 9.3 and 8.2 ka events.

The regional significance of the LoNAP514 δ^13^C hydroclimatic record is attested by a comparison with lacustrine carbonate δ^18^O changes from the FC and Anatolia^45–50^ (Fig. 3).

Eastern Mediterranean/Anatolian lacustrine δ^18^O is mostly driven by the precipitation/evaporation ratio and, similarly to speleothems, lower/higher δ^18^O ratios usually indicate higher/lower precipitation^51^. Within the combined uncertainties, and accounting for diverse time-resolution, a very similar multi-centennial pattern is apparent, especially within the 10.0–8.0 ka interval (Fig. 3). Between 9.7 ± 0.2 and 9.0 ± 0.1 ka, all records coherently express wetter conditions. This is followed by an abrupt reduction of precipitation between 9.0 and 8.5 ± 0.1 ka, and by a wetter interval between 8.5 and 8.0 ± 0.2 ka (Fig. 3). The high temporal resolution of the LoNAP514 δ^13^C also reveals superimposed, lower-amplitude, centennial-scale moisture fluctuations. Reduced precipitation occurred between 9.3 ± 0.2 and 9.1 ± 0.1 ka, at 8.9 ± 0.1 ka, between 8.1 ± 0.2 and 7.9 ± 0.2 ka, and at 7.1 ± 0.2 ka. Considering the associated age uncertainties, the first and the third of these events are consistent, in terms of both duration and timing, with the 9.3 and 8.2 ka climatic anomalies described above. However, they are not particularly prominent (Fig. 3). This suggests that the downstream atmospheric changes related to these RCCs had only a limited impact on local precipitation, and that there are other drivers for the observed climatic variability.

## Teleconnections and mechanisms of hydroclimate variability

**Figure 4:**
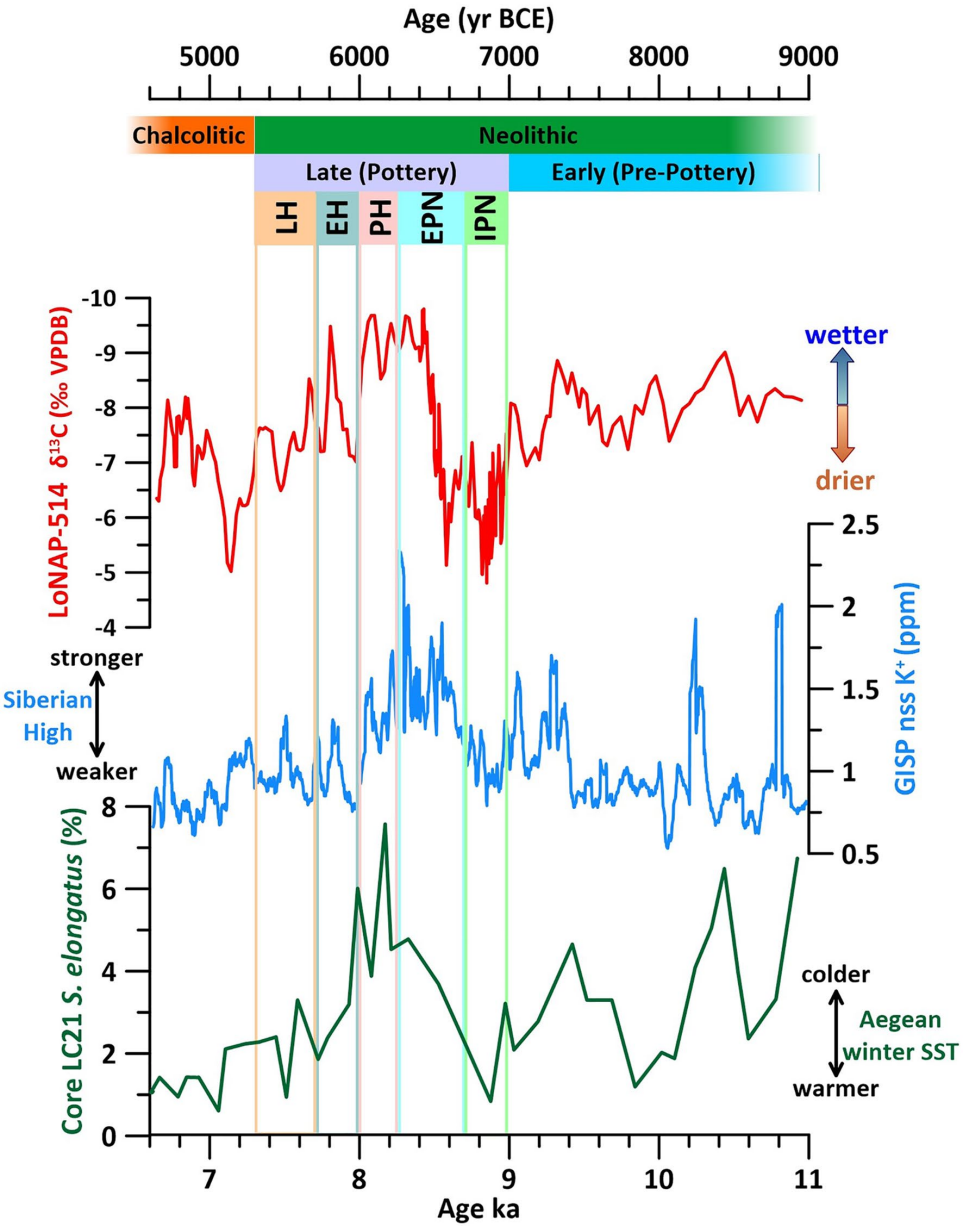
From bottom: Percentage of cold dynocist *Spiniferites elongatus* from the Aegean core SL21^43^ 15 points-smoothed nssK + from Greenland ice core GISP2^53^; δ^13^C (this study); Major cultural phases identified in the northern FC^3,26^ (IPN: Initial Pottery Neolithic; EPN: Early Pottery Neolithic; PH: Pre-Halaf; EH: Early Halaf; LH: Late Halaf).

Despite differences in the long-term (millennial) trends, the LoNAP514 precipitation record closely mimics centennial-scale fluctuations in the non-sea-salt potassium (nssK^+^) record from the Greenland GISP2 ice core^52^, with higher precipitation at the cave site corresponding to increases in nssK^+^ content (Fig. 4). Such increases have been correlated to enhanced dust transport from Asia, related to an intensified Siberian High (SH) in winter/spring^52^. This implies a strong teleconnection between centennial-scale fluctuations in the intensity of the SH and precipitation in the northern FC during the early to mid-Holocene. Notably, the similarity is better expressed between ca. 10 and 8 ka, where the SH is at its strongest (Fig. 4). Currently, seasonal and inter-annual rainfall variability in the FC is explained mostly by changes in the intensity, frequency and orientation of eastern Mediterranean storm tracks and by changes in the supply of water vapor from southern sources^31^. Thus, to understand the mechanisms modulating precipitation patterns in our record, we need to consider the influence of the SH on both Mediterranean and southern synoptic conditions.

**Figure 5:**
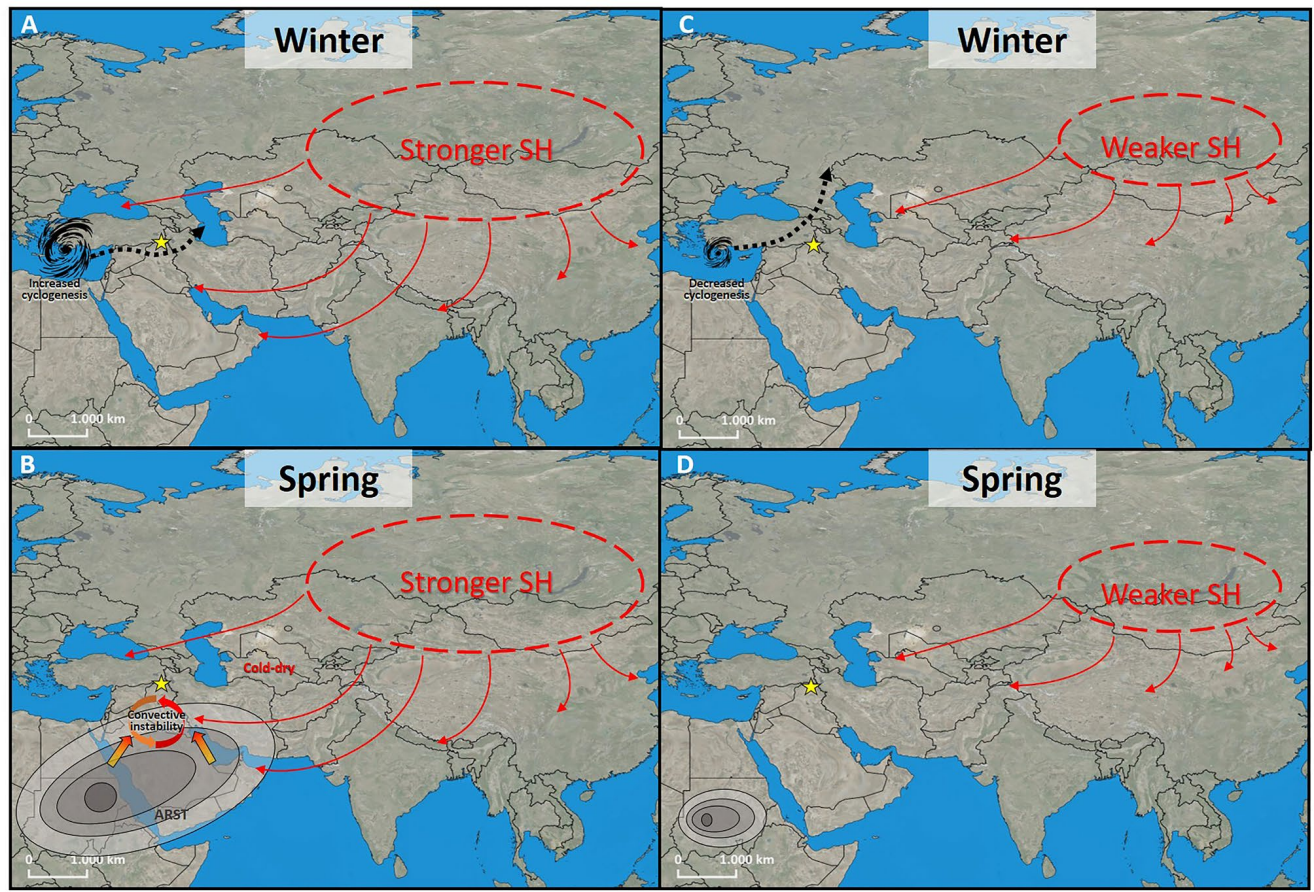
Schematic of the proposed mechanisms influencing precipitation in Northern Iraq. For stronger (**A**, **B**) and weaker (**C**, **D**) Siberian High on winter (**A**–**C**) and spring (**B**–**D**) conditions. The star is the LoNAP514 cave site. Red arrows represent the influx of cold-dry air from the SH; the black dotted arrows in panels (**A**–**C)** represent the trajectories of the Eastern Mediterranean storm tracks; the thick red-yellow arrows in panel B represent the low-tropospheric southerly influx of warm and moist air. ARST = Active Red Sea Trough (contracted to Sudan Low in panel). See text for explanation. All maps are elaborated with QGIS 3.16.7 plugin https://nextgis.com/blog/quickmapservices/.

Current observations and modelling show that a strengthening of the SH increases the frequency and intensity of cold northerly air outbreaks that are channeled through gaps in the mountain ranges along the north-eastern Mediterranean borderlands^54^. These northerly fluxes deliver cold/dry air masses to the warm/moist Aegean Sea sector, promoting intense evaporation and creating or intensifying local cyclogenesis. This causes extreme precipitation events in the Levant, exceptional snowfall around the Aegean region, and flash floods in the Middle East^54^. Cluster analyses of eastern Mediterranean cyclones also shows that the intensification of the SH influences storm-track trajectories. In particular, a blocking configuration over central Asia and eastern Europe favors a direct eastward route toward southern Anatolia and the northern FC, instead of the usual north-eastward trajectories to the north of the Black Sea^55^. Looking at the paleo-record, the centennial to multi-centennial influence of the SH-induced cold outbreaks on Eastern Mediterranean conditions can be demonstrated by the consistent timing between increases in the GISP nsK + content and sea-surface temperature (SST) cooling events in the Aegean Sea^19,43^. Specifically, changes in the abundance of the cold-water dinocyst *Spiniferites elongatus* from core SL21 have been used to identify relative winter SST changes through the early to middle Holocene in the central Aegean^43^. The LoNAP514 precipitation record shows strong similarities with the *S. elongatus* record, with colder winter conditions in the Aegean corresponding to higher precipitation (Fig. 4). Reference^19^ modelled the effect of northerly outbreaks in the Aegean on the local hydroclimatic cycle and found that their increased frequency and intensity cause strong evaporation in the eastern Aegean, enhancing the atmospheric moisture content over the sea. When these moist air masses subsequently make landfall and are cooled and/or uplifted, their relative humidity increases and precipitation may develop, leading to cooler and wetter conditions over the Levant. Despite our site being located further away from the Mediterranean coast than the Levant, the similarity with the Aegean record suggests that a critical component for the increase in precipitation in the northern FC during intervals of enhanced SH is the effect of cold northerly outbreaks in promoting eastern Mediterranean evaporation and cyclogenesis. In particular, an intensified cyclonic circulation increases the advection of moist air towards the north/northeast, whereas the westward expansion of the SH causes a southward shift in storm tracks^55^ (see also sketch on Fig. 5). These combined effects likely increased the amount of winter precipitation in the northern FC during early Holocene intervals of intensified SH, especially between 9.7 and 9.0 ka and between 8.5 and 8.0 ka.

Regarding the influence of the SH on southerly vapor supply and synoptic conditions, it has been found that the increase of SH strength is one of the dynamic factors generating Active Red Sea Troughs (ARST)^56^. ARST originates from the Sudan Low, which is a part of the large-scale subtropical/equatorial low-pressure thermal system known as the intertropical convergence zone (ITCZ). ARST development results from the interaction between a lower-tropospheric inflow of warm, moist air of tropical origin extending northward from the southern Red Sea toward the eastern Mediterranean and the Middle East, and a mid-tropospheric southward inflow of cold and dry air from Eurasian mid-latitudes^56^. Such conditions are associated with unstable stratification and a high concentration of vapor sourced from the Persian Gulf/Arabian Sea reaching the FC. This favors the development of highly energetic mesoscale convective systems, which can produce intense precipitation and flooding, especially at the fringe of high-relief regions, such as along the SW Zagros flanks^57^. Regional climate models simulate convective precipitation maxima in transitional months, especially in April–May, when enough energy is present to trigger local convection, but before the summer season, when high pressure related to the descending branch of the Hadley Cell begins to dominate the area^31^. The position of the Hadley Cell is directly related to the seasonal movement of the ITCZ, that also determines the onset, duration and termination of the Indian summer monsoon (ISM)^58^. In the last 150 years, the date of ISM onset has varied between May 11 (1918) and June 18 (1972)^59^. ISM onset is modulated by large-scale forcing, such as the land-sea thermal contrast between the Eurasian landmass and the Indian Ocean. A delayed onset is usually associated with colder spring conditions over the Tibetan Plateau and negative SST anomalies in the tropical and subtropical oceans during the season prior to the monsoon onset (i.e., March–May)^60,61^. Winter-spring temperatures over central and East Asia are negatively correlated with the strength of the SH^28,62^, and a relationship have been found between a stronger SH and negative SST anomalies in the Indian Ocean^63^. Furthermore, at present, the intensity of the SH and the strength of the Hadley circulation exhibit a significant negative correlation both for their inter-annual variability and for their secular trend^63^. Therefore, we propose that, across the 9.7–9.0 and the 8.5–8.0 ka intervals, the strengthening of the SH was responsible for increased evaporation and advection from southerly source, and for increased convective instability related to the formation of a more frequent/stronger ARST (Fig. 5). At the same time, a stronger SH also caused a delayed onset of the ISM due to negative temperature anomalies that persisted longer in spring over the Eurasian land masses and in the sub-tropical oceans. The two combined effects boosted the occurrence of convective precipitation, and allowed their development to persist well within May, causing a net increase in spring precipitation over the FC. After 8.0 years BP, the Northern Hemisphere ice sheets were reduced and the SH weakened^9^, leading to reduced convective instability across the Middle East, and to warmer spring conditions across Eurasia, preventing the delayed onset of the ISM, reducing the length of the wet season in the FC and leading to a divergence between the long-term trends of the SH and of precipitation at our site (Fig. 4).

## Archaeological implications

**Figure 6:**
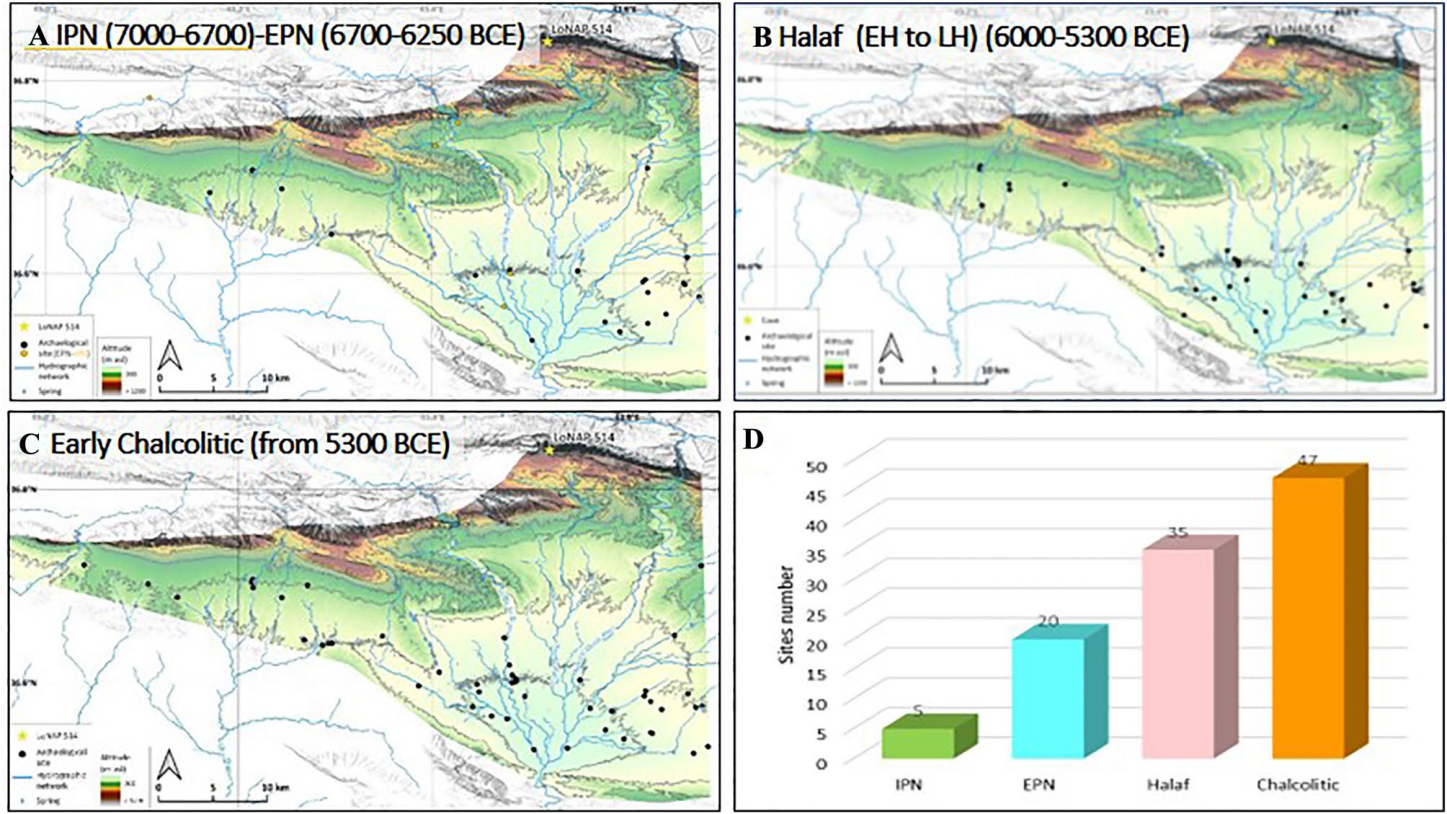
(**a**–**c**): Maps illustrating the distribution of archaeological sites identified by LoNAP survey and their relationship with water resources. The different panels cover the different cultural phases discussed in the text (basemaps are derived from DEM free downloaded from https://www.eorc.jaxa.jp/ALOS/en/aw3d30/ and elaborated with QGIS 3.16.7). (**d**) graph showing the number of sites for the different periods.

In the last decade, a wealth of data from surveys and excavations in the northern FC have deepened the knowledge on regional Neolithic and early Chalcolithic cultural traits and settlement patterns (i.e., size, distribution and duration of occupation), and of their interrelationships with landscape and environmental resources^3,64,65^. Updated summaries of the latest regional evidence anchored to a comprehensive radiocarbon-dated framework are now available^3,26^, providing a detailed regional archaeological background that allows for a robust comparison with the hydroclimatic variability revealed by the LoNAP514 record. Furthermore, systematic archaeological survey has been performed by the LoNAP project (see Acknowledgement) specifically in the study region (i.e. along the Zagros foothills and the left Tigris River tributaries; Fig. 6), leading to the identification of a number of archaeological sites that were attributed to different cultural phases within the PN mostly through analyses of pottery types. The LoNAP survey data for the PPN are instead scarce, mostly because PPN sites are challenging to locate, either because they can be covered by later levels at tell sites, buried by alluvial sediments, or because their conservation is hampered by natural or anthropogenic erosion. Thus, they are not discussed here.

Thanks to this new evidence, inferences can be made regarding relationships between settlements and the key factor for their survival by way of water resources, whose availability is linked to precipitation patterns. During the latest stage of the Early (Pre-Pottery) Neolithic (PPN), populations in the northern FC took full advantage of a rich environment, pursuing a flexible mix of subsistence strategies. These included hunting and foraging of wild game and plants, as well as herding a small number of species, especially goats^5^, and cultivating a range of pulse and cereal crops in fields on the surrounding plains^3^. Regionally, sites are widespread in both the plains and the foothills; they are usually small (less than 1 ha) and often appear to be only seasonally occupied^3^. Over this period, the LoNAP514 record shows relatively wet conditions (Fig. 4) that would have allowed the first forms of rain-fed and/or décrue cultivation during the rainy season^66^.

At the regional scale, the initial phase of the Pottery Neolithic (IPN, 9.0–8.7 ka; 7000–6700 BCE)^29^ and references therein] witnessed a contraction of the inhabited sites, with most of the settlements located in the upper part of the valleys, and above of the present-day 220 mm isohyet. All these sites fall close to major and perennial watercourses^65^. This contraction corresponds precisely to the marked reduction in precipitation apparent from the LoNAP514 record (Fig. 4), suggesting that climatic factors hampered the expansion of settlements in the IPN and influenced their location. The early Pottery Neolithic (EPN, 8.7–8.3 ka, 6700 to 6250 BCE)^26^ saw the full uptake of pottery techniques^26^. During the EPN, settlements were remarkably short-lived and had a highly mobile nature, and the number of settlements increased, thus suggesting a noticeable demographic growth^26^. A gradual spread from the upper part of the valleys to more downvalley locations is apparent. Specifically in the study area, survey data shows that the EPN is marked by the first systematic exploitation of the lowlands along main streams (Fig. 6a). Sites still appear to cluster around watercourses, either permanent or seasonally active, whereas no EPN sites have been found on the semi-arid steppe separating them. In particular, a detailed analysis of settlement distribution shows that they were located mostly within 100–200 m of a watercourse^65^. Over this period, the LoNAP514 record shows the transition from drier conditions (up to 8.5 ka, 6500 BCE) towards a marked increase in precipitation characterized, according to our interpretation, by a longer rainy season with a potential increase in extreme convective events. This scenario fits well with the regional archaeological evidence^6,26^: in the initial phase of the EPN, the adoption of more durable pottery containers likely facilitated the collection of surpluses over longer times, giving a net advantage under drier conditions and allowing the inferred demographic rise. Subsequently, wetter climate conditions supported the growth and the new spreading of human groups^6^ to locations that were abandoned during the IPN, and along (now) seasonal streams, that at the time were likely active for most of the year. However, due to the increased frequency of extreme precipitation events, sites were located at a certain distance from the rivers, preventing the risk of settlement flooding but close enough to exploit inundated land for seasonal cultivation^65^.

The period between 8.3 and 7.3 ka corresponds to the Pre-Halaf (PH; 6250–6000 BCE) and (Early to Later) Halaf cultural phases (EH, 6000–5700 BCE; LH 5700–5300, Fig. 4)^26^. During the PH at regional scale, site distribution still resembled that of the preceding EPN, with small sites (1–2 ha) concentrated along rivers and streams^26^. Further, the LoNAP514 record does not highlight significant changes leading up to 8.0 ka. Subsequently, with the onset of the Halaf phase at ~ 6000 BCE (i.e., from ~ 8 ka), regional synthesis shows settlement expansion, and dispersion of some communities into the steppe. This pattern is also evident from survey data in the study region, with the distribution of Halaf settlements suggesting the progressive occupation of inter-river areas (Fig. 6b). This new use of the territory is part of a multifaceted package of economic, technological, and cultural innovations and adaptations (e.g., full adoption of administrative systems and an intensified reliance on secondary products) that profoundly transformed the Late Neolithic societies and increased their complexity^26^. Throughout the EH, people appear to move around the landscape dynamically, founding new settlements, relocating to others, and abandoning them easily^26,67^. Furthermore, these communities also adopted flexible subsistence strategies relying on a combination of agriculture and herding a range of domesticated plants and animals, but also on hunting and foraging of wild species. In all likelihood, this residential and subsistence flexibility made the best of challenging environments, such as those of the semi-arid steppe^26^. The subsequent LH witnessed a surge in the number of sites, and their continuous spreading into regions far from water resources. Settlement size and internal complexity appear to increase towards the end of the Halaf phase, with the largest sites (up to at least 10 ha) located near perennial water courses^26,65^. However, recent interpretations that take account of the dynamism of frequent relocation apparent for the northern FC suggest that the Halafian “mega-site” can be better explained as a small-scale settlement shifting over favourable spots in the local landscape across several generations^26^. According to our record, the EH and LH phases occurred in the context of the change in precipitation amount and style towards more arid climatic conditions between 8.0 and 7.7 ka (6000–5700 BCE), and in the subsequent highly variable period up to 7.3 ka (5300 BCE), respectively (Fig. 4). This coincidence suggests that many of the transformations observed in the archaeological record across this period may have been expedited by climatic stress but were mostly triggered by technological and cultural innovations.

A dynamic response to changing background climatic conditions is even more apparent in the subsequent Early Chalcolithic phase, from ~ 5300 BCE (~  7.3 ka): in this time span, villages evolved towards increased complexity; the same applies to their social organization. Furthermore, in the whole FC, the total number of sites appears to increase markedly during the Early Chalcolithic^6^. Interestingly, the shift from the late Neolithic to the Chalcolithic, marked in our record by a quite drastic precipitation reduction (Fig. 4), appears also characterized by a change in the use of water resources, with many sites located close to perennial springs and rivers in the Zagros Mountain piedmont zone. This regional trend is confirmed by local survey data, which also highlight the increase in settlement numbers in the Chalcolithic and a change in the strategy of exploitation of permanent water resources (Fig. 6c). This suggests that the climatic deterioration fostered the use of previously poorly exploited resources well before the onset of irrigated cultivation in the Bronze Age.

In summary, the integration of high-resolution climate records with robust, contextualised archaeological data is a crucial strategy for better understanding both the climatic variability itself and its potential societal consequences^68^. Our inference regarding the influence of the SH on precipitation amount and seasonality in the FC brings clarity to a rich collection of climate proxy records in the region that indicate significant variability, but lack a clear temporal coherency with—or an univocal expression of—the major Early Holocene RCCs (the 9.3 and the 8.2 ka events). In fact, our record does not support a direct influence of Early Holocene RCCs on the process of Neolithization and its cultural dispersal. Although it is still debated whether or not Neolithic cultural changes were forced directly by climatic variability, we show robust chronological agreement between changes in precipitation pattern and the alternation of local cultural phases, suggesting that hydroclimate variability influenced the way in which Neolithic population exploited the surrounding environment. This is particularly significant in terms of settlement strategies and use of water resources. In this view, climate variability, leading to increased stress or amelioration of background environmental conditions, appears to accelerate and force existing cultural and subsistence dynamics, which were however not directly attributed to the climatic change itself^8,66^. Climate variability thus acts as a stimulus that continuously interacted with technological and cultural adaptations of complex societies^69,70^, fostering the evolution of new and multifaceted adaptive strategies that progressively increased the resilience of ancient populations and modified the way they interacted with their environment.

## Methods

Subsamples for δ^13^C and δ^18^O were drilled at a mean resolution of 0.3 mm and analyzed using a Precision AP2003 continuous-flow isotope-ratio mass spectrometer at the School of Geography, Earth and Atmospheric Sciences at the University of Melbourne (UoM) using the method reported in Hellstrom ^71^. Mean analytical uncertainties are 0.07‰ and 0.05‰ for δ^18^O and δ^13^C, respectively. U–Th dating was performed at the School of Geography, Earth and Atmospheric Sciences (UoM) on 17 calcite prisms of ~ 100 mg, employing a multi-collector inductively coupled plasma mass spectrometer (MC-ICP-MS; Nu-Instruments Plasma) and the method presented in Hellstrom^72^. The age-depth model (Supplementary Information, Fig. S2) has been obtained by Monte Carlo modelling and stratigraphic constrains following^71^. Systematic survey of archaeological evidence has been carried out within the LoNAP framework by applying ‘extensive methodology’, that is, via analysis of contemporary as well as historical satellite/aereal images followed by direct ground-truthing through direct field walking; details can be found in refs^64,65^.

## Supplementary Information

Supplementary Information 1.

Supplementary Information 2.

## Data Availability

Data presented in this work (U–Th dating and stable isotopes) are available as Online Supporting Information (Tables S1 and S2 respectively).
