# Supplementary material for: Neolithic hydroclimatic change and water resources exploitation in the Fertile Crescent
Source: Sci Rep. 2023 Jan 13;13:45. doi: 10.1038/s41598-022-27166-y (PMC9839760; doi:10.1038/s41598-022-27166-y)
Supplement: Supplementary file 2 — Supplementary Information 2. [file 41598_2022_27166_MOESM2_ESM.docx]

**Supplementary Information for**

**Neolithic hydroclimatic change and water resources exploitation in the Fertile Crescent**

**Text 1: Geological and geomorphological background**

Specimen LoNAP514 was retrieved at ⁓15 m from the entrance of a shallow cave (36°50'24.87"N 43°31'2.95"E) located in the part of the Zagros Mountains traversing the governorate of Dohuk, in the northern Kurdistan Region of Iraq. The region is part of the Zagros-Fold Thrust Belt (ZFTB) formed by continental collision - active since the Early Miocene - between the Arabian and Eurasia plates (Csontos et al., 2012; Dewey et al., 1973; Dercourt et al., 1986; Fouad, 2015; Mouthereau et al., 2012). Deformation of the Zagros Mountains has propagated over the NE margin of the Arabian Plate towards the Mesopotamian Foreland Basin and the Persian Gulf (Blanc et al., 2003; Csontos et al., 2012; Vergés et al., 2011). In the study area, the ZFTB is organized into four diﬀerent zones, moving from the inner part of the orogeny (Imbricated and High Fold zones) to its foreland (Foothills zone and Mesopotamian Foreland Basin) (Berberian, 1995; Fouad, 2015; Frizon de Lamotte et al., 2011; Jassim and Goﬀ, 2006). The deformation created a series of anticline folds that represent the main structural features of the local landscape (Forti et al., 2021). In the Dohuk area, anticlines are W–E trending, while in the eastern and southern sector of the mountain belt they are NW–SE oriented. Geological strata folded and deformed in this section of the ZFTB include Upper Triassic/Lower Cretaceous to Pliocene units. Lithologically, the local bedrock includes Ordovician sandstones and Carboniferous-Permian limestones and shale, Upper Triassic to Upper Cretaceous reef limestone and dolomitic limestone with inter?bedded marls and shales, Eocene limestone (Sissakian, 2014; Zebari et al., 2019), Upper Paleocene to Lower Miocene limestone, dolostone and sandstone, and Plio-Pleistocene conglomerates. From the geomorphological point of view, the anticlines are widely spaced, and the synclines create swales and plains ﬁlled by various Quaternary sediments, including alluvial fans and ﬂoodplain deposits, which are occasionally deformed by recent tectonic activity. Tectonic uplift and exhumation rate of the anticlinal ridges and syncline troughs promoted the formation of different physiographic units, resulting in a complex landscape (Forti et al., 2021). The large plain opening at the foothills of the anticlines is crossed by the Tigris River and its many left-bank tributaries, whose watersheds extend along the Zagros Mountains. Today, most of the streams show seasonal-to-ephemeral activity, and only the Tigris and its major tributaries are permanent. In the rainy season, the discharge of streams increases and flooding along the plains is common.

**Figure S1:**

**
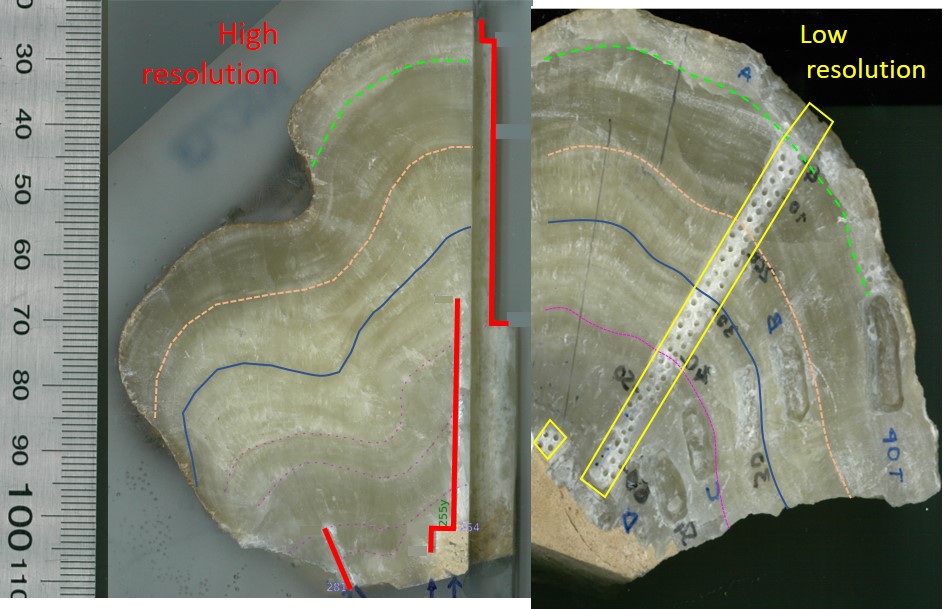
**

Figure S1. The sampled pieces of flowstone LoNAP514 reporting the stable isotope sampling tracks (low in yellow and high resolution in red). The low-resolution sampling was done manually using an hand-held drill (Dremel) and a 1 mm drill-bit. The high-resolution sampling was performed with a milling machine with an average resolution of 0.3 mm. Samples for dating were cut from the step resulting from the high-resolution sampling. Coloured thin lines represent identified laminations used to match the high- and low-resolution isotope series.

**Figure S2:**


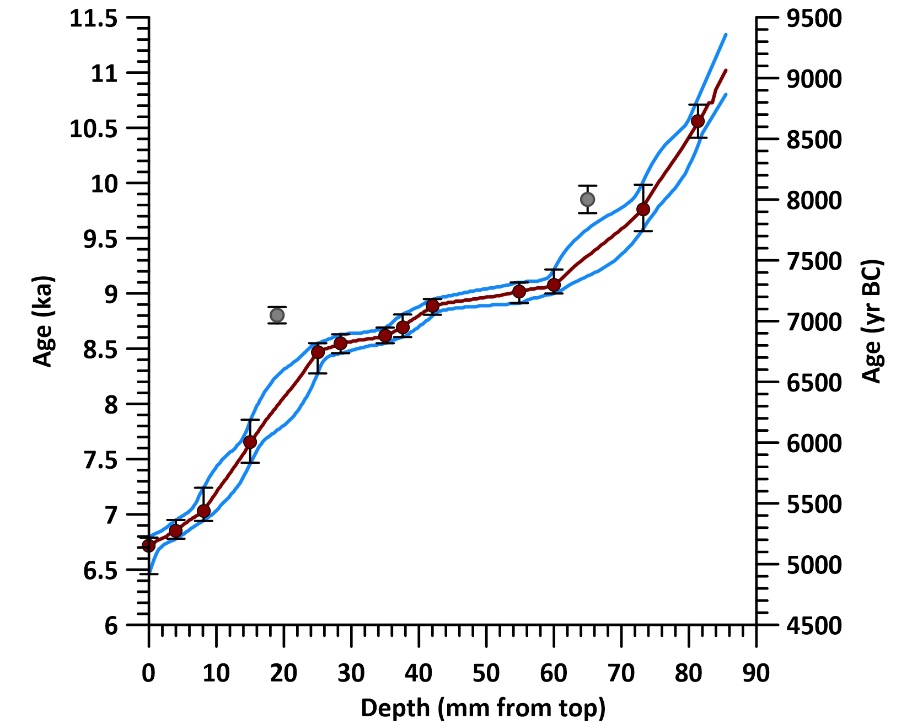


Figure S2. Age-depth model for flowstone LoNAP514. Ages shown in grey were rejected as outliers. The age-depth model was constructed following Drysdale et al. (2005) and Hellstrom et al. (2006).

**Text 2: Statistical comparison of δ^13^C and δ^18^O isotope ratios**

To quantify similarities between the two isotope series we apply the procedure previously described by Regattieri et al. (2014). We first smoothed the two isotope series using a 3-points moving average. Then we normalize each series (by using their mean and standard deviation as normalization parameters) to produce a correspondent time series of anomalies (i.e., deviations from a zero mean expressed in standard deviation units). The standard deviation between the two normalized series was then calculated and plotted as a grey shadow, together with the two individual anomaly series, in Fig. 2 of the main text.

**Text 3: Equilibrium deposition**

For flowstone LoNAP514, the assumption of quasi isotope equilibrium deposition of calcite was tested using two lines of reasoning. First, thin section microstratigraphy reveals that the dominant fabric is compact columnar calcite (Fig. S3). In sparitic speleothem, columnar fabric and its subtypes form with relatively constant drip discharge and low calcite supersaturation state, and are commonly associated with quasi-equilibrium deposition (Frisia, 2015; Frisia and Borsato, 2010).

Second, from LoNAP514, two different stable isotope series were retrieved (low and high spatial resolution). These followed two different sampling tracks located in different portions of the same flowstone (Fig. S1). The close coherency between the two series (Fig. S3) testify the absence of significant kinetic fractionation, which would have resulted in different isotope patterns in different parts of the flowstone. Indeed, when deposition occurs close to equilibrium, almost constant values of δ^18^O and δ^13^C along a single growth layer are observed (Hendy, 1971). The small discrepancies between the two series may indicate minor kinetic effects (that are always apparent in speleothems: Daëron et al., 2019) and/or the influence of slighlty different growth rates for the two lobes of the flowstone from which the series were retrieved, but these differences are small compared to the overall range of values.


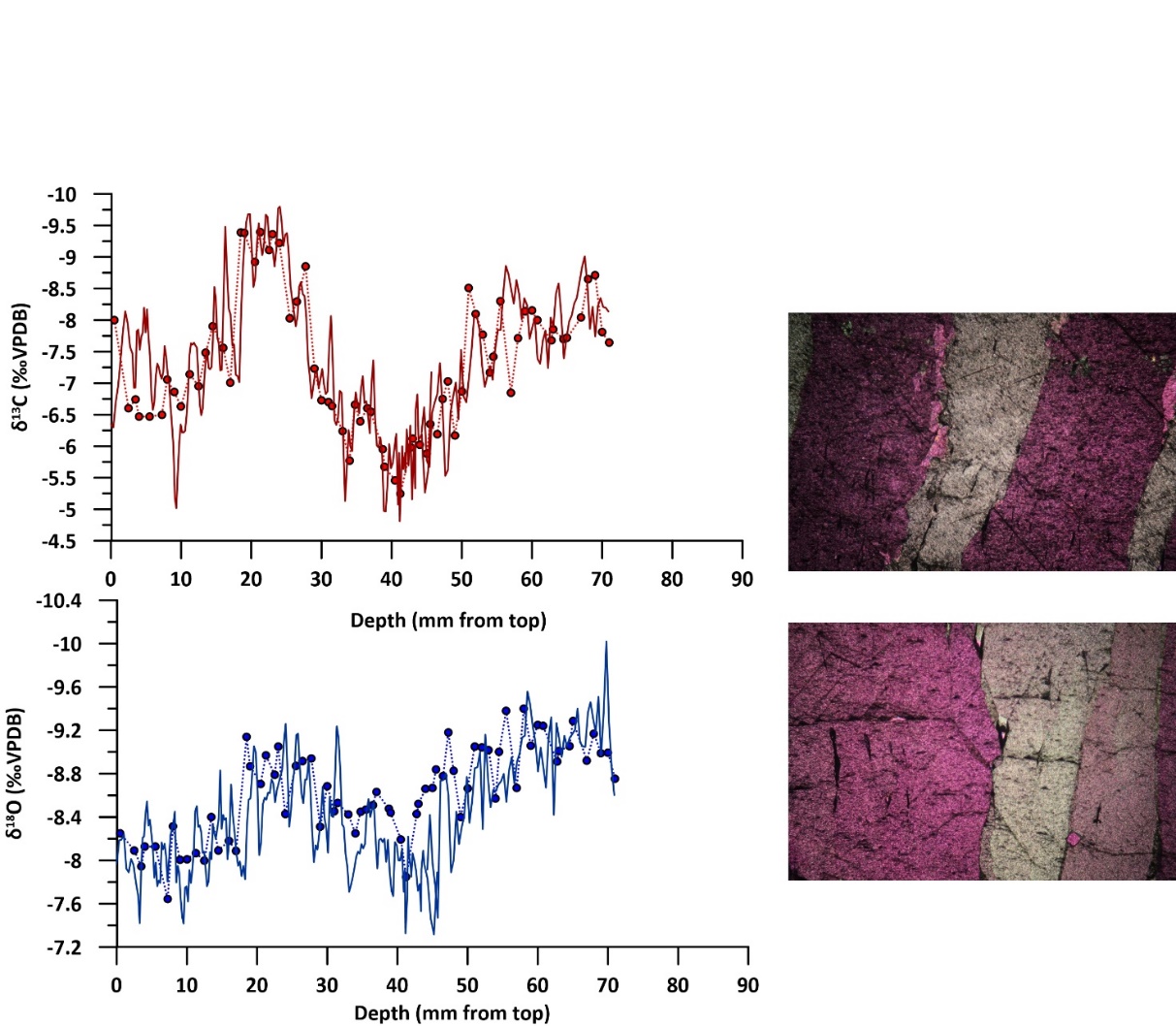


Figure S3: Left panel: Low (dotted) vs. high (solid) resolution stable isotope time series for flowstone LoNAP514 plotted versus depth. Right panel: Thin section microphotographs showing compact columnar calcite (cross polarized nicols, base of the photo 5.2 mm).

**Text 4: Palaeoclimatic interpretation of LoNAP514 δ^18^O**

Assuming quasi-isotopic equilibrium calcite precipitation, the speleothem δ^18^O is a proxy for drip-water δ^18^O, which reflects local meteoric precipitation δ^18^O (δ^18^O_p_) and eventually further solution modification occurring in the soil and in the epikarst (mostly evaporation) (e.g. Baker et al. 2019). Presently, at the annual scale, northern FC δ^18^Op is strongly influenced by both the condensation temperature (with a 0.79‰ increase for 1°C of increasing temperature) and the amount effect (with a depletion of 2.3‰ for 100 mm of increase in precipitation) (Fig. S4).


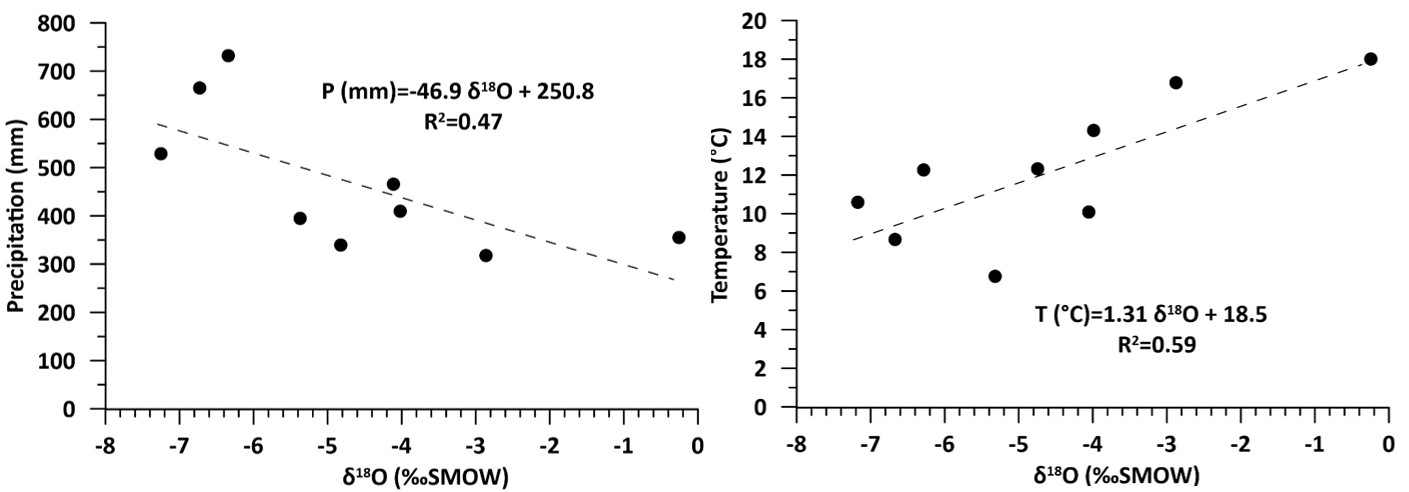


Figure S4: Correlation between precipitation δ^18^O and precipitation amount (left) and temperature (right) in west Iran-East Iraq; re-drafted from Mohammadzedeh et al., 2020

This means that both temperature changes and precipitation fluctuations influence, in an opposite fashion, the δ^18^Op. The effect of these gradients on the forming speleothem calcite can be difficult to disentangle. However, despite condensation temperature changes can be important when the seasonal or annual scale is considered, at the time scale considered here (i.e. centennial to multicentennial) the effect of changes in the amount of rainfall appear to be dominant on the final speleothem δ^18^O, as testified by the strong covariance with the δ^13^C record (see also main text and SI text 5).

At the time scale considered here, another important factor affecting the speleothem δ^18^O is the effect of changes in the isotopic composition of the moisture source. The Eastern Mediterranean is the main source of precipitation for the Northern FC, and its surface water δ^18^O (δ^18^O_sw_) is known to be influenced by changes in freshwater discharge (i.e. ^18^O depleted) by the Nile River, related to changes in monsoonal activity recharging the upper Nile catchment. Holocene changes in EMed δ^18^Osw are recorded -at low-resolution- by the δ^18^O record of planktonic foraminifera *G. ruber* in cores LC21 (Marino et al., 2009; Fig. S5). Despite some mismatch between the age models (related to the higher uncertainty of the marine record) the comparison with the LoNAP514 δ^18^O record shows the highest similarities when the standard deviation between the two speleothem isotope series is higher (Fig. S5). A more detailed view on the influence of the source effect on the LoNAP δ^18^O can be obtained by the comparison with the record of Fe/Ca from core MS27PT, recovered directly at the Nile mouth (Revel et al., 2010). Here, changes in Fe/Ca are considered a proxy for changes in terrigenous input from the Nile, with increases in the Fe/Ca ratio related to increased fluvial discharge (Fig. S5). For this comparison is apparent that some of the centennial-scale negative spikes observed in the oxygen record have a close analogue in the detrital record, indicating the influence of short-time freshwater pulses on the isotopic composition of precipitation reaching the FC.

Overall, the comparison with the E Mediterranean record shows that the speleothem δ^18^O is, at various time scales, influenced by changes in the isotopic composition of the source of precipitation, that may be unrelated to precipitation variability in the cave area. Considering that this “source effect” does not influence the speleothem δ^13^C record, the latter can be considered a more robust proxy for hydroclimatic change in the cave area.


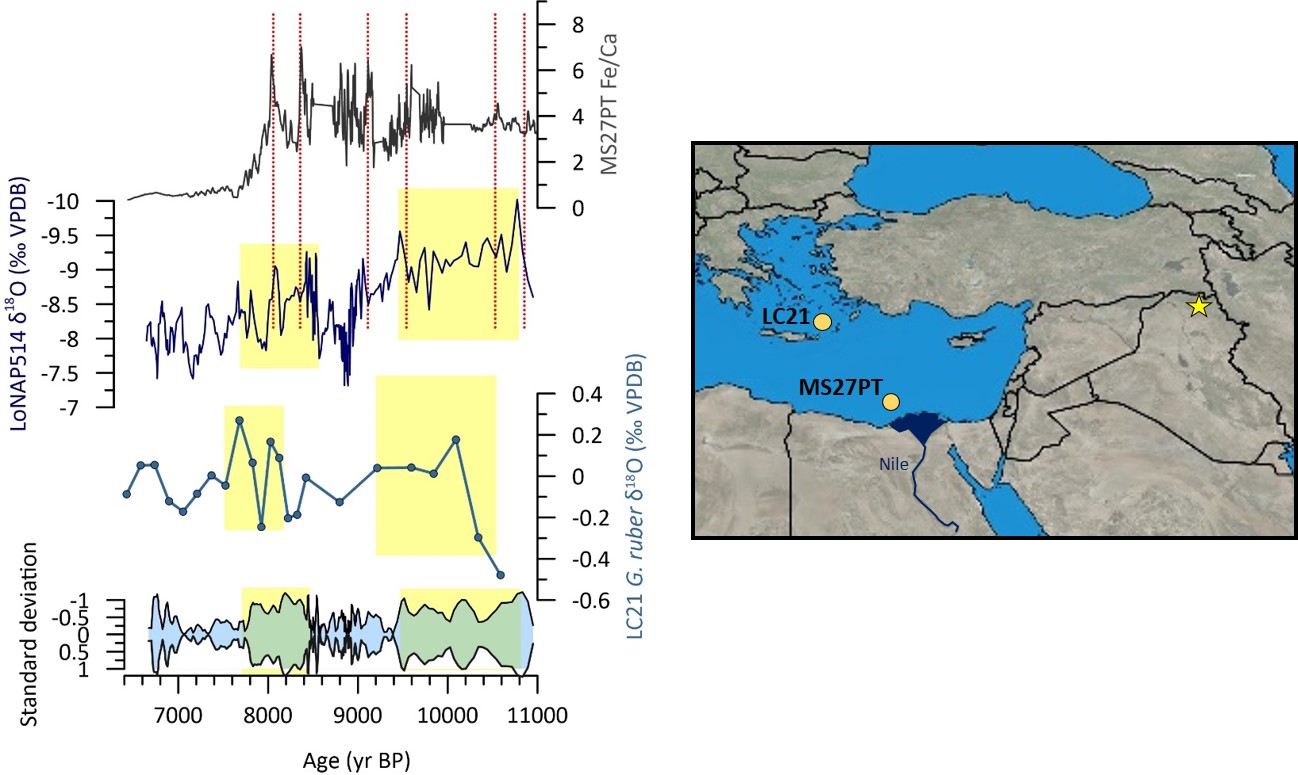


Fig. S5: Left panel, from bottom: standard deviation between normalized δ18O and δ13C record for LoNAP514; δ18O record of planktic foraminifera Globigerinoides ruber in core LC21 (Marino et al., 2009); LoNAP514 δ18O record (this study); Fe/Ca ratio from core MS27PT (Revel et al., 2010). Yellow rectangles mark the intervals where the standard deviation between the speleothem isotope series is higher (corresponding to stronger similarities between the speleothem and the planktic δ^18^O series); red dotted lines highlight negative spikes in the speleothem δ18O and correspondent peaks in terrigenous input from the Nile. Right panel: location of cores LC21 and MS27PT, the star is the LoNAP514 cave site.

**text 5: Holocene vegetation in the cave area and influence on the δ^13^C composition of LoNAP514**

Today, the vegetation cover in the cave area is sparse and mostly represented by seasonal wild grasses. However, this is unlikely to represent a direct analogue for the Early to Mid-Holocene vegetation due to long-lasting human land-use for agriculture and pastoralism, which deeply altered the natural vegetation and caused impoverishment and erosion of the soil cover (e.g., Roberts et al., 2002; 2011).

Most of the Zagros Mountains area is covered by ‘Kurdo-Zagrosian steppe-forest’, which is composed of three major vegetation types, depending on the altitudinal belt. In the mid-altitudes (1800-1200 m a.s.l.), deciduous oak woodland occurs, dominated by the xerophilous *Quercus brantii* that has expanded in the region since the Mid-Holocene (Djamali et al., 2010; Roberts, 2002). In the lower altitude belt (1200-750 m a.s.l.), to which the cave catchment belongs, the dominant vegetation is represented by *Pistacia-Amygdalus* scrubland. In the lower (below 750 m a.s.l), drier altitudes, the Irano-Turanian Artemisia steppes in the east (central Iran) and the Mesopotamian lowland savannas in the west, represents the most common vegetation (Zohary, 1973). Other species typical of the medium altitude vegetation are *Pyrus syriaca, Crataegus aronia, Cerasus microcarpa, Acer monspessulanum, L. subsp. cinerascens, Amygdalus scoparia* (Browicz and Zielinski, 1982). All these species belong to the C3 vegetation type.

Soil δ^13^C at C3 vegetation sites is expected to be in the range -26‰ to -20‰ (Rudzka et al., 2011; Cerling et al., 1991). Assuming a simplified system working near isotopic equilibrium at each stage, it is possible to obtain a raw estimation of the δ^13^C of speleothem calcite in equilibrium with a labile soil carbon pool by adding ⁓10‰ to the value of soil CO_2_ (Rudzka et al., 2011). This produces a range of -16‰ to -10‰ for the calcite. The range of δ^13^C values observed for LoNAP514 is slighlty lower (⁓-5 to -10‰). This difference could be related to an incomplete equilibration between soil CO_2_ and the dissolved inorganic carbon (DIC), resulting in a more pronounced contribution of ^13^C-enriched CO_2_ from bedrock dissolution (Bajo et al., 2017; Rudzka et al., 2011; Hendy et al., 1971). However, due to the thin bedrock above the cave where LoNAP514 was retrieved, this effect is likely negligible for our sample. Lowering of drip rates, increased cave ventilation, and water evaporation in the soil and in the epikarst - all capable of promoting preferential degassing of ^12^CO_2_ - can also result in enriched δ^13^C values. Of these, the latter yields strong covariation with the δ^18^O (because also ^16^O would be preferentially evaporated), but does not imply significant kinetic fractionation during calcite precipitation (as the other effects do). Because equilibrium conditions appear to be maintained throughout the deposition of LoNAP514 owing to its consistent fabric, we therefore consider evaporation in the soil and in the epikarst as the main factor causing the enriched ^13^C composition of LoNAP514 (see also the main text). Furthermore, it is noteworthy that all the above mentioned effects shift the isotopic signal in the same direction, driving the speleothem δ^13^C toward less negative ratios when the climate is drier. This further supports the use of LoNAP514 δ^13^C ratios as the most reliable hydroclimatic proxy.

**SI Appendix references**

Bajo, P., Borsato, A., Drysdale, R., Hua, Q., Frisia, S., Zanchetta, G., Hellstrom, J.C., Woodhead, J., 2017. Stalagmite carbon isotopes and dead carbon proportion (DCP) in a near-closed-system situation: an interplay between sulphuric and carbonic acid dissolution. Geochimica and Cosmochimica Acta 210, 208-227.

Baker, A., Hartmann, A., Duan, W., Hankin, S., Comas-Bru, L., Cuthbert, M. O., Treble, P. C., Banner, J., Genty, D., & Baldini, L. M. (2019). Global analysis reveals climatic controls on the oxygen isotope composition of cave drip water. Nature Communications, 10(1), 2984.

Berberian, M. (1995). Master “blind” thrust faults hidden under the Zagros folds: Active basement tectonics and surface morphotectonics. Tectonophysics, 241(3–4), 193–224.

Blanc, E. J.-P., Allen, M. B., Inger, S., Hassani, H. (2003). Structural styles in the Zagros simple folded zone, Iran. Journal of the Geological Society, 160(3),

Browicz K, Zieliński J (1982) Chorology of trees and Shrubs in South-west Asia and Adjacent Regions. Volume 1. Polish Scientific Publishers.

Cerling, T.E., Solomon, D.K., Quade, J.A.Y., Bowman, J.R., 1991. On the isotopic composition of carbon in soil carbon dioxide. Geochem. Cosmochim. Acta 55 (11), 3403-3405.

Csontos, L., Sasvári, Á, Pocsai, T., Kósa, L., Salae, A. T., Ali, A. (2012). Structural evolution of the northwestern Zagros, Kurdistan region, Iraq: Implications on oil migration. GeoArabia, 17, 81–116

Daëron, M., Drysdale, R.N., Peral, M., Huyghe, D., Blamart, D., Coplen, T.B., Lartaud, F., Zanchetta, G., 2019. Most Earth-surface calcites precipitate out of isotopic equilibrium. Nature Communication 10 (1), 1-7.

Dercourt, J., Zonenshain, L. P., Ricou, L.-E., Kazmin, V. G.,Le Pichon, X., Knipper, A. L., Grandjacquet, C., Sbortshikov, I. M., Geyssant, J., Lepvrier, C., Pechersky,D. H., Boulin, J., Sibuet, J.-C., Savostin, L. A.,Sorokhtin, O., Westphal, M., & Bazhenov, M. L. (1986). Geological evolution of the tethys belt from the atlantic to the pamirs since the LIAS. Tectonophysics, 123(1-4), 241–315

Dewey, J. F., Pitman, W. C., Ryan, W. B. F., Bonnin, J. (1973). Plate tectonics and the evolution of the alpine system. Geological Society of America Bulletin, 84(10), 3137– 3180.

Djamali, M., Akhani, H., Andrieu-Ponel, V., Braconnot, P., Brewer, S., de Beaulieu, J. L., Fleitmann, D., Fleury, J., Gasse F., Guibal F., Jackson S.T., Lézine A-M., Médail, F., Ponel, P., Roberts, N.C., Stevens, L. (2010). Indian Summer Monsoon variations could have affected the early-Holocene woodland expansion in the Near East. The Holocene, 20(5), 813-820.

Drysdale, R.N., Zanchetta, G., Hellstrom, J.C., Fallick, A.E., Zhao, J.X., 2005. Stalagmite evidence for the onset of the Last Interglacial in southern Europe at 129±1 ka.mGeophys. Res. Lett. 32 (24).

Forti, L., Perego, A., Brandolini, F., Mariani, G. S., Zebari, M., Nicoll, K., Regattieri, E., Barbaro C.C., Bonacossi, D.M., Qasinm H.A., Cremaschi, M., Zerboni, A. 2021. Geomorphology of the northwestern Kurdistan Region of Iraq: landscapes of the Zagros Mountains drained by the Tigris and Great Zab Rivers. Journal of Maps, 1-12.

Fouad, S. F. A. (2014). Western Zagros fold – Thrust Belt, part II: The high folded zone. Iraqi Bulletin of Geology and Mining, 6, 53–71.

Frisia, S., 2015. Microstratigraphic logging of calcite fabrics in speleothems as tool for palaeoclimate studies. International Journal of Speleology 44 (1), 1-16.

Frisia, S., Borsato, A., 2010. Karst. Development in Sedimentology 61, 269-318.

Frizon de Lamotte, D., Raulin, C., Mouchot, N., Wrobel-Daveau, J. C., Blanpied, C., Ringenbach, J. C. (2011). The southernmost margin of the tethys realm during the mesozoic and cenozoic: Initial geometry and timing of the inversion processes. Tectonics, 30(3).

Hellstrom, J., 2006. UeTh dating of speleothems with high initial 230Th using stratigraphical constraint. Quat. Geochronol. 1 (4), 289-295

Hendy, C.H., 1971. The calculation of the effects of different modes of formation on the isotopic composition of speleothems and their applicability as palaeoclimatic indicators. Geochimica et Cosmochimica Acta 35, 801–824.

Jassim, S. Z., Goff, J. C. (2006). Geology of Iraq. First ed. (341 pp.). Dolin Prague and Moravian Museum.

Marino, G., Rohling, E. J., Sangiorgi, F., Hayes, A., Casford, J. L., Lotter, A. F., et al. (2009). Early and middle Holocene in the Aegean Sea: interplay between high and low latitude climate variability. Quaternary Science Reviews, 28(27-28), 3246-3262.

Mohammadzadeh, H., Eskandari Mayvan, J., & Heydarizad, M. (2020). The effects of moisture sources and local parameters on the 18O and 2H contents of precipitation in the west of Iran and the east of Iraq. Tellus B: Chemical and Physical Meteorology, 72(1), 1-15.

Mouthereau, F., Lacombe, O., Vergés, J. (2012). Building the Zagros collisional orogen: Timing, strain distribution and the dynamics of Arabia/Eurasia plate convergence. Tectonophysics, 532-535, 27–60.

Revel, M., Ducassou, E., Grousset, F. E., Bernasconi, S. M., Migeon, S., Révillon, S., Mascle, J., Murat, A., Zaragosi, S., Bosch, D. (2010). 100,000 Years of African monsoon variability recorded in sediments of the Nile margin. Quaternary Science Reviews, 29(11-12), 1342-1362.

Roberts, N., Eastwood, W. J., Kuzucuoğlu, C., Fiorentino, G., & Caracuta, V. (2011). Climatic, vegetation and cultural change in the eastern Mediterranean during the mid-Holocene environmental transition. The Holocene, 21(1), 147-162.

Hendy, C.H., 1971. The calculation of the effects of different modes of formation on the isotopic composition of speleothems and their applicability as palaeoclimatic indicators. Geochimica and Cosmochimica Acta 35, 801e824.

Regattieri E., Zanchetta G., Drysdale R., Isola I., Hellstrom J., Dallai, L. 2014. Lateglacial to Holocene trace element record (Ba, Mg, Sr) from Corchia Cave (Apuan Alps, central Italy): paleoenvironmental implications. Journal of Quaternary Science, 29(4), 381-392.

Roberts, N., Eastwood, W. J., Kuzucuoğlu, C., Fiorentino, G., & Caracuta, V. (2011). Climatic, vegetation and cultural change in the eastern Mediterranean during the mid-Holocene environmental transition. The Holocene, 21(1), 147-162.

Roberts N (2002) Did prehistoric landscape management retard the post-glacial spread of woodland in Southwest Asia? Antiquity 76: 1002–1010.

Rudzka, D., McDermott, F., Baldini, L.M., Fleitmann, D., Moreno, A., Stoll, H., 2011. The coupled d13C-radiocarbon systematics of three Late Glacial/early Holocene speleothems; insights into soil and cave processes at climatic transitions.

Sissakian, V. K. (2014). Geological Map of Arbeel and Mahabad Quadrangles Sheets NJ-38-14 and NJ-38-15, Scale 1 V 250:000

Vergés, J., Saura, E., Casciello, E., Fernandez, M., Villasenor, A., Jimenez-Mount, I., Garcia-Castellanos, D. (2011). Crustal-scale cross-sections across the NW Zagros belt: Implications for the Arabian margin reconstruction. Geological Magazine, 148(5-6), 739–761

Zebari, M., Grützner, C., Navabpour, P., Ustaszewski, K. (2019). Relative timing of uplift along the Zagros Mountain front flexure (Kurdistan region of Iraq): constrained by geomorphic indices and landscape evolution modeling. Solid Earth, 10(3), 663–682.

Zohary M (1973) Geobotanical Foundations of the Middle East. 2 volumes. Gustav Fischer Verlag.
